# Association Between Body Mass Index and Comorbid Anxiety in First-Episode and Drug Naïve Patients With Major Depressive Disorder

**DOI:** 10.1155/da/6648190

**Published:** 2025-05-10

**Authors:** Shilin Liu, Yu Huang, Aixin Liu, Xiaoxuan Li, Yifan Fu, Wei Wang, Yuechun Wen, Tao Jiang, Xiangyang Zhang

**Affiliations:** ^1^Department of Neurosurgery, The First Affiliated Hospital of Anhui Medical University, Hefei, Anhui 230001, China; ^2^Anhui Public Health Clinical Center, Hefei, Anhui 230001, China; ^3^Department of Ophthalmology, Provincial Hospital Affiliated to Anhui Medical University, Hefei, Anhui 230001, China; ^4^Department of Orthopaedic Surgery, Lu'an Hospital of Traditional Chinese Medicine Affiliated of Anhui University of Traditional Chinese Medicine, Lu'an, Anhui 237000, China; ^5^Hefei Fourth People's Hospital, Anhui Mental Health Center, Affiliated Psychological Hospital of Anhui Medical University, 316 Huangshan Road, Shushan District, Hefei, Anhui Province 230022, China

**Keywords:** anxiety symptoms, body mass index, double robust estimation, major depressive disorder, non-linear relationship

## Abstract

**Objective:** There is limited evidence regarding the relationship between body mass index (BMI) and anxiety symptoms in individuals with major depressive disorder (MDD), and the findings are controversial. This study aimed to explore the association between BMI and anxiety symptoms in patients with first-episode drug-naïve (FEDN) MDD.

**Methods:** A total of 1718 FEDN MDD patients were included in this study, gathering information on their sociodemographic attributes and physical measurements. BMI was classified into three categories (normal, overweight, and obese) based on the standards of the Working Group on Obesity in China (WGOC). Logistic regression and double robust estimation were used to assess the association between anxiety symptoms and BMI. Additionally, a restricted cubic spline (RCS) analysis was used to examine the relationship between anxiety symptoms and BMI. If nonlinear associations existed, threshold effects were analyzed using a two-piecewise logistic regression model. The subgroup analysis was performed to validate the robustness of the findings.

**Results:** Among 1718 patients, 12.7% (218) exhibited anxiety symptoms. After adjusting for confounding variables, multivariable logistic regression analysis revealed a positive association between BMI and the risk of experiencing anxiety symptoms (OR = 1.13, 95% CI: 1.039–1.229, *p*=0.004). These findings were further confirmed using a doubly robust estimation. Additionally, RCS analysis revealed a nonlinear correlation between BMI and anxiety symptoms, with a turning point of 26.9 kg/m^2^. On the left side of the inflection point, a positive association between BMI and anxiety symptoms was detected (OR = 1.167, 95% CI: 1.055–1.296, *p*=0.003), while no significant association was observed on the right side of the inflection point (OR = 1.01, 95% CI: 0.685–1.341, *p*=0.972). Subgroup analyses revealed significant variations in the association between gender and education level.

**Conclusion:** This study demonstrated that a higher BMI was associated with an increased risk of experiencing anxiety symptoms in Chinese patients with FEDN MDD, particularly among those with a BMI below 26.9 kg/m^2^.

## 1. Introduction

Major depressive disorder (MDD) is a psychological disorder characterized by ongoing feelings of depression, impaired concentration, decreased appetite, insomnia, constant tiredness, and, in severe cases, thoughts or actions of self-harm [1]. Anxiety is a common clinical subtype of depression, characterized by major depression and significant anxiety burden [2, 3]. Previous research revealed that individuals with MDD who also experience comorbid anxiety exhibit elevated suicidal rates, increased behavioral and psychiatric symptoms, prolonged illness duration, reduced responsiveness to antidepressant treatment, and fewer favorable outcomes compared to those without anxiety [4–7]. Most patients with MDD and anxiety are treated with antidepressants, which are also given to MDD patients without anxiety [8]. Recent research has demonstrated that anxious depression is linked to more significant immune dysregulation, cortex thinning, and cortical limbic system dysfunction than depression [9].

Metabolic dysregulation is commonly observed in individuals with MDD, frequently manifesting as alterations in body mass index (BMI), including weight loss and obesity [10]. Research has indicated a potential association between BMI deviations and an elevated MDD risk concomitant with anxiety disorders, although the precise nature of this relationship remains unclear [11]. For instance, individuals with an increased BMI may encounter increased physiological stress, including metabolic disorders and hormonal fluctuations, which could potentially augment their susceptibility to anxiety and depressive symptoms [12, 13]. Conversely, individuals with low body weight may experience increased anxiety due to metabolic or nutritional deficiencies, complicating their mental health challenges [14]. Moreover, metabolic factors, including altered cortisol levels, which are common in individuals with abnormal BMI, may influence the onset or exacerbation of anxiety symptoms in MDD [15, 16].

Recent neuroimaging studies have provided further insights into this relationship. For example, functional magnetic resonance imaging (fMRI) has demonstrated that brain regions like the prefrontal cortex and amygdala may exhibit altered activity in individuals with higher BMI, potentially contributing to elevated anxiety symptoms [17]. Furthermore, neurobiological studies have suggested that changes in neurotransmitter levels and hormonal fluctuations, which are often associated with obesity, could affect the stress response system of the brain, thereby exacerbating anxiety and depressive symptoms [18]. However, the mechanisms linking BMI and anxiety are complex and multifactorial.

Anxiety is often linked to alterations in stress hormones, including cortisol, which can contribute to increased appetite and weight gain in some patients [19]. Conversely, severe anxiety and increased sympathetic activity can suppress appetite, potentially resulting in weight loss [20]. This relationship is complicated by several factors, including ethnicity, clinical characteristics, and treatment history, hindering the establishment of a clear causal pathway. First, most studies on the relationship between BMI and anxiety symptoms in patients with MDD have focused on Western populations, with limited data from Asian and Chinese populations. This is particularly relevant given the differences in BMI distributions, cultural attitudes toward body weight, and genetic predispositions between Western and Eastern populations. Second, many studies have included patients with varying stages of depression (for instance, acute, chronic, or remitted) and those who have undergone different treatment regimens, which may confound the relationship between BMI and anxiety symptoms.

Chinese patients with first-episode drug-naïve (FEDN) MDD might provide a distinct opportunity to explore the association between BMI and concurrent anxiety symptoms without the impact of lifestyle alterations and medications. To the best of our knowledge, the link between BMI and anxiety symptoms in Chinese patients with FEDN MDD has not yet been investigated. This study aimed to investigate the relationship between BMI and anxiety symptoms in Chinese patients with FEDN MDD.

## 2. Methods

### 2.1. Subjects

From September 2016 to December 2018, all patients were sourced from the outpatient psychiatric department of a general hospital in China. Two seasoned psychiatrists independently assessed all patients. The inclusion criteria were as follows: (1) fulfilling the Diagnostic and Statistical Manual of Mental Disorders, Fourth Edition, Text Revision (DSM IV-TR) criteria for MDD; (2) having a first episode of current depressive symptoms; (3) having no previous treatment with antidepressants; (4) Han Chinese individuals aged between 18 and 65 years; (5) except smoking tobacco, no other substance abuse or dependency. The exclusion criteria included the following: (1) presence of severe physical illnesses, including chronic infections, cancer, epilepsy, brain injury, or stroke; (2) diagnosis of other major Axis I disorders, including schizophrenic affective disorder, schizophrenia, bipolar disorder, or other psychotic conditions; (3) pregnant and lactating female patients; (4) patients declining to sign informed consent documents. Ultimately, 1718 eligible patients were included in this study. This study was approved by the Ethics Committee of the Shanxi Medical University's First Clinical Medical College (No. 2016-Y27).

### 2.2. Demographic Characteristics

Basic information, including age, gender, weight, height, education level, marital status, and duration of illness, was collected using a self-designed, standardized questionnaire. BMI was calculated as the ratio of body weight (kg) to the square of height (m^2^), and nutritional status was assessed according to the criteria set by the Working Group on Obesity in China [21]. Because only a few participants (*n* = 10) had a BMI < 18.5 kg/m^2^, the underweight individuals were grouped with those having normal BMI. BMI was classified as standard (<24 kg/m^2^), overweight (24 ≤ BMI < 28 kg/m^2^), and obese (≥28 kg/m^2^) [22, 23].

Depressive symptoms were assessed using the Hamilton Rating Scale for Depression (HAMD). The scale includes eight items scored from 0 (absent) to 4 (severe) and nine items rated from 0 (absent) to 2 based on symptom-specific severity. A total HAMD-17 score ≥ 24 was used as the diagnostic criterion for MDD [24, 25].

Anxiety symptom severity was evaluated using the Chinese version of the Hamilton Anxiety Rating Scale (HAMA), which comprises 14 symptom-specific items. Each item is rated on a 5-point Likert scale ranging from 0 (absent) to 4 (severe), yielding a total score range of 0–56. A cutoff score of 25 was applied to classify participants into groups with or without severe anxiety symptoms [26, 27].

Psychotic symptoms were assessed using the Positive and Negative Syndrome Scale (PANSS) positive subscale. Each item was rated on a scale of 1 (absent) to 7, with a total score of 7–49. A total subscale score of 15 or higher was used to define the presence of psychotic symptoms [28, 29].

Two experienced psychiatrists with at least 5 years of clinical practice were trained to administer these rating scales. Repeated assessments demonstrated a correlation coefficient above 0.8, indicating strong inter-rater reliability.

### 2.3. Blood Samples

Blood samples were collected from all participants via venipuncture between 6:00 and 9:00 a.m., following an overnight fast. The samples were processed at the hospital laboratory on the same day and analyzed before 11:00 a.m. The levels of thyroglobulin antibody (TgAb), thyroid peroxidase antibody (TPOAb), free T4 (FT4), and free T3 (FT3) were assessed using a Roche C6000 electrochemiluminescence immunoassay analyzer. The ARCHITECT c8000 System was used to measure blood lipid profiles, including triglycerides (TG), cholesterol (TC), high-density lipoprotein cholesterol (HDL-C), and low-density lipoprotein cholesterol (LDL-C).

### 2.4. Independent Variables and Covariates

Different BMI levels were used as independent variables. Variables significant in univariate analyses or clinically relevant were used to modify the covariates in subsequent multivariate analyses. Statistical analyses and clinical judgment revealed that age at onset, gender, duration of illness, education, HAMD, LDL-C, HDL-C, TG, TC, fasting blood glucose (FBG) levels, FT3, TgAb, and TPOAb were confounding factors.

### 2.5. Statistical Analyses

The normality of the data was assessed using the Agostino test, followed by descriptive analysis. Continuous variables are expressed as mean with standard deviation for parametric data, while nonparametric data are presented as median with interquartile range. Differences between groups were assessed using a one-way analysis of variance (ANOVA) or the Kruskal–Wallis test. Categorical variables are expressed as frequencies and percentages, and comparisons were made using the chi-square or rank-sum test.

BMI was analyzed as a continuous and categorical variable (classified based on predefined BMI cutoffs), and its linear association with anxiety symptoms was evaluated using logistic regression models. The findings are presented as unadjusted and adjusted odds ratios (ORs) with 95% confidence intervals (CIs). Three models were developed to assess the robustness of the findings. Model 1 was analyzed using univariate analysis without any covariate adjustments. Model 2 included all the 13 previously mentioned factors. Model 3 used a stepwise backward selection method based on the Akaike information criterion. Collinearity was assessed using the variance inflation factor (VIF). Variables with a VIF > 5 were excluded from the model [30].

Double robust estimation models were used to confirm these results. The propensity scoring model includes 13 covariates, and the propensity scores were calculated using logistic regression. The observations across BMI groups were reweighted using inverse probability treatment weighting (IPTW), creating three comparable groups for all variables [31, 32]. Subsequently, weighted regression analysis was conducted on all the propensity scoring model confounders, yielding double robust estimators for each BMI group.

The nonlinear effect of BMI was further investigated using restricted cubic spline analysis to address potential information loss and changes in the dose–response relationship when categorizing continuous variables [33]. In cases where a nonlinear relationship was identified, the threshold value was determined using the recursive algorithm from the scitb5 function, and a two-segment linear regression model was applied on either side of the identified inflection point to study the association between BMI and anxiety symptoms.

Besides, subgroup analyses were performed based on gender and education to investigate the strata effect and potential interactions. All statistical analyses were performed using the R software (http://www.r-project.org, The R Foundation). The primary R package used in this study included the “foreign” package, “ggplot2” package, “ggrcs” package, “Hmisc” package, “ipw” package, “scitb5” package, “rms” package. The “scitb5” package was used to determine the threshold value. The threshold for statistical significance was set at *p* < 0.05.

## 3. Results

### 3.1. Sociodemographic Characteristics

This study included 1718 patients diagnosed with FEDN MDD, with a mean age of 34 years (range: 23–45). The sample comprised 34.2% males (588/1718) and 65.8% females (1130/1718). Anxiety symptoms were observed in 218 patients, accounting for 12.7% of all participants. The baseline characteristics of participants, categorized by BMI index tertiles, are presented in Table 1. Significant correlations were found between BMI index tertiles and multiple variables, including anxiety symptoms (*p* < 0.01), age (*p*=0.005), length of illness (*p*=0.009), onset (*p*=0.005), marital status (*p* < 0.05), TC (*p*=0.002), TG (*p*=0.038), FBG (*p* < 0.001), and TSH (*p* < 0.001).

### 3.2. Associations Between Anxiety Symptoms and in Various BMI Index

**Figure 1:**
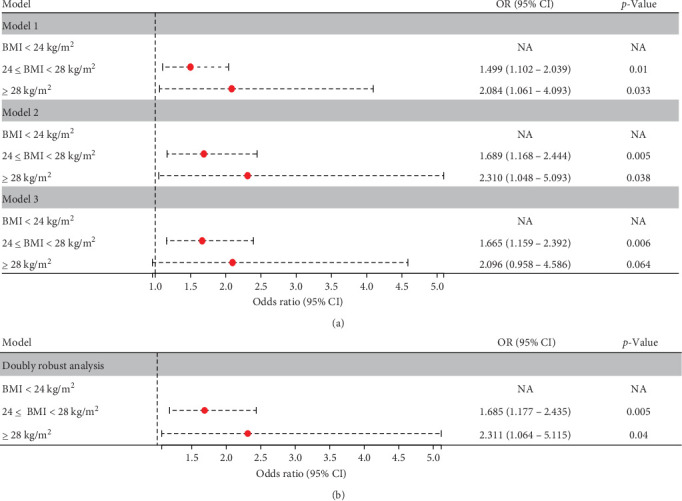
(A) the results of univariate and multivariate logistic regression when analysed the influence of varying BMI levels on the occurrence of anxiety symptoms in FEDN MDD patients; Model 1 represented the univariate analysis; Model 2 represented the multivariate analysis adjusting all covariates; Model 3 represented the multivariate analysis adjusting duration of illness, HDL-C, TPOAb, TC and HAMD based on the results of stepwise backward approach and collinearity analysis. (B) the results of the univariate logistic analysis conducted following the double robust estimation.

The connection between BMI and anxiety symptoms was examined using univariate logistic regression models.  Table 2 includes the results of both the unadjusted and adjusted models. Three statistical models, each accounting for different confounders, showed that the ORs and 95% CI for both overweight and obesity groups were significantly above 1.0, suggesting a higher occurrence of anxiety symptoms compared to those with a normal BMI index group. In Model 1 (univariate analysis), the overweight group exhibited an OR of 1.499 (95% CI: 1.102–2.039; *p*=0.01), while the obesity group showed a higher OR of 2.084 (95% CI: 1.061–4.093; *p*=0.033). After adjusting for all 13 covariates in Model 2, the ORs (95% CI) were 1.689 (1.168–2.444) for the overweight group and 2.310 (1.048–5.093) for the obesity group, with *p*-values of 0.005 and 0.038, respectively. In the fully adjusted logistic regression model, the ORs (95% CI) for the overweight and obesity groups were 1.665 (1.159–2.392) (*p*=0.006) and 2.096 (0.958–4.586) (*p*=0.064), respectively.  Figure 1A shows that all models displayed a similar trend. Notably, the risk of anxiety symptoms was comparable between the overweight and obese groups.

### 3.3. Double Robust Estimation

IPTW was used to equalize the baseline characteristics among the three BMI groups before performing double robust estimation. IPTW, based on multinomial logistic regression, effectively addressed the significant imbalance in the original data.

To assess the incidence of anxiety symptoms, a double robust estimation was performed, adjusting for all 13 covariates included in the IPTW. Compared to the normal BMI group, the overweight group showed a significantly higher risk of anxiety symptoms (OR = 1.685; 95% CI 1.177–2.435, *p*=0.005), while the obesity group exhibited an even greater risk (OR = 2.311; 95% CI 1.064–5.115, *p*=0.04) (Figure 1B).

### 3.4. Nonlinear Relationship Analysis

**Figure 2:**
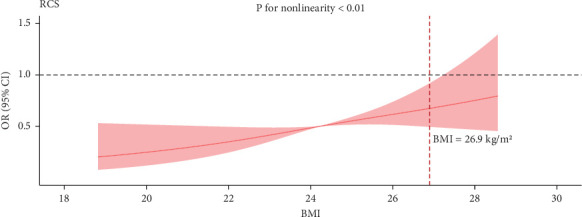
Association between BMI and anxiety symptoms. Multivariable adjusted odds ratios for anxiety symptoms according to initial BMI on a continuous scale was a nonlinear relationship (*p* for nonlinearity < 0.01). Solid red lines were multivariable-adjusted odds ratios, with red areas showing 95% confidence intervals derived from restricted cubic spline regressions with five knots. Reference lines for no association were indicated by the black dashed lines at a odds ratio of 1.0 and the reference knot setted at 26.9 kg/m^2^. All 13 covariates were adjusted.

After adjusting for age at onset, gender, duration of illness, education, HAMD, TC, TG, HDL-C, LDL-C, FBG, FT3, TGAb, and TPOAb, Figure 2 illustrates the non-linear association between BMI and anxiety symptoms (*p* for non-linearity < 0.01; *p* for log-likelihood ratio test < 0.05). A two-piecewise logistic regression model revealed an inflection point at a BMI value of 26.9 kg/m^2^. For each unit increase in BMI on the left side of the inflection point, the likelihood of anxiety symptoms increased by 16.7% (OR = 1.167, 95% CI: 1.055–1.296, *p*=0.003). Conversely, on the right side of the inflection point, no significant association was observed between BMI and anxiety symptoms (OR = 1.006, 95% CI: 0.685–1.341, *p*=0.972), as detailed in Table 3. The cohort included 129 patients with a BMI of 26.9 kg/m^2^ or higher and 1589 patients with a BMI below 26.9 kg/m^2^.

#### 3.4.1. Subgroup Analysis

**Figure 3:**
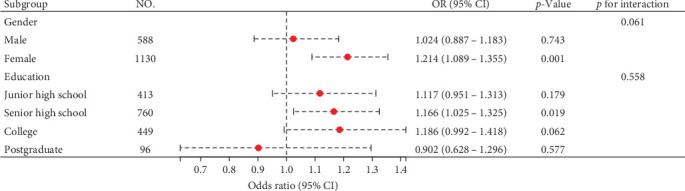
Subgroup analysis of the association between BMI and anxiety symptoms. The logistic regression model derived the OR (95% CI). All were adjusted for age at onset, gender, duration of illness, education, HAMD, TC, TG, HDL-C, LDL-C, FBG, FT3, TGAb, and TPOAb.

Figure 3 illustrates the subgroup analysis results. Interaction tests with gender and education were non-significant for anxiety symptoms (*p*=0.061 and 0.558). However, analysis revealed a significant impact of the BMI index on females (OR = 1.214, 95% CI: 1.089–1.355, *p* < 0.001) and individuals with senior high school degrees (OR = 1.166, 95% CI: 1.025–1.325, *p*=0.019).

## 4. Discussion

Multiple analytical approaches were used in this study, including logistic regression, IPTW, and double robust estimation, to assess the relationship between anxiety symptoms and BMI in a large cohort of individuals diagnosed with FEDN MDD. The results revealed a robust association between BMI and anxiety symptoms, which remained significant after adjusting for potential confounders. There was a nonlinear relationship between BMI and anxiety symptoms, with an inflection at 26.9 kg/m^2^. On the left side of the inflection point, BMI was positively correlated with anxiety symptoms. Simultaneously, no statistically significant relationship was observed on the right side of the inflection point. Furthermore, these effects were exacerbated among females and individuals with senior high school degrees.

These findings align with previous research indicating that obese or overweight individuals are predisposed to psychological distress and anxiety symptoms [11, 14, 34]. This study advances this field of research by elucidating the nonlinear relationship between BMI and anxiety symptoms, particularly in patients experiencing first-episode anxiety who have not undergone medication intervention. This approach minimized the confounding effects of medication and lifestyle factors. Moreover, the positive relationship between BMI and anxiety symptoms remained statistically significant, even after adjusting for age at onset, gender, illness duration, education, HAMD, TG, TC, HDL-C, LDL-C, FBG, FT3, TGAb, and TPOAb. Consistent with the findings of this study, Luo et al. [11] reported a significant association between BMI and comorbid depression and anxiety. Notably, overweight and obese patients exhibit more severe anxiety symptoms, particularly women. In an age-stratified adjusted analysis, some researchers [14] observed that anxiety was not positively correlated with obesity in adults.

However, several studies have reported conflicting results. In a large cross-sectional study, Zhao et al. [35] discovered that American men with a BMI ≥ 40 kg/m^2^ were more likely to experience acute or lifetime depression and anxiety than those with a normal BMI. Similarly, men with BMI < 18.5 kg/m^2^ exhibited a higher risk of lifetime depression. Among women, both overweight and obese individuals were at a higher risk of developing all three psychiatric disorders than those with a normal BMI. A longitudinal study discovered that individuals with a current comorbid depressive severity and anxiety disorder exhibited a U-curved weight association during the 2-year follow-up [36]. In individuals with lower body weight, elevated anxiety levels may be attributed to nutritional deficiencies and imbalances in neurotransmitter functions. Conversely, individuals with normal weight often exhibit lower anxiety levels, which may be associated with a more favorable metabolic state and reduced weight-related pressures. However, anxiety risks increase as body weight progresses to obesity. Adipose tissues are now recognized as more than an energy storage site; they function as active endocrine organs, releasing various metabolically active adipocytokines. These adipocytokines are associated with neurohormonal processes and metabolic dysregulation, influencing systems, including the sympathetic nervous system, the renin–angiotensin-aldosterone axis, and leptin regulation [37]. Anxiety, which is caused by excessive worrying and physical symptoms due to increased sympathetic nervous system activation, is also influenced by plasma leptin levels, which affect stress, anxiety, and sadness [38, 39]. Moreover, this phenomenon may be attributed to the detrimental effects of obesity on self-esteem, particularly in the context of limited sociocultural support and inadequate awareness regarding body image concerns. This combination of factors may predispose individuals to numerous anxiety symptoms, including panic attacks and social phobia, resulting from increased body dissatisfaction and psychological distress. This U-shaped relationship illustrates the bidirectional influence of BMI on mental health, demonstrating the importance of maintaining a healthy weight for psychological well-being. In this study, the nonlinear association between BMI and anxiety symptoms suggests that, below a threshold of 26.9 kg/m^2^, a further increase in BMI does not confer additional benefits in reducing anxiety symptoms.

The variations observed in the relationship between BMI and anxiety symptoms across studies may be influenced by several factors. First, cultural and ethnic distinctions, including lifestyle, values, beliefs, and genetic predispositions, may contribute to discrepancies between the Western and Eastern populations. Second, differences in antidepressant use among participants might play a role, as antidepressants vary not only in their efficacy in treating depression but also in their influence on anxiety symptoms. Third, sample heterogeneity in previous research, such as variation in disease stages (acute versus remission) and types of depressive disorders, could influence findings. Finally, the definition of anxiety symptoms varied across studies. For instance, the STAR(*⁣*^*∗*^)D study defined anxious depression as a HAMD-17 anxiety/somatization subscale score ≥7 in patients with MDD [3]. Subsequent studies have assessed anxiety severity in patients with MDD using the HAMD-17 anxiety/somatization subscale. In this study, the HAMA scale, a more targeted measure of anxiety symptoms, was used, providing a reliable and valid indicator of anxiety severity in depressed patients [40].

Conversely, studies have revealed that patients with MDD and anxiety experience more severe symptoms, more frequent episodes, and BMI variations as anxiety levels change [35, 41, 42]. Anxiety is often associated with fluctuations in stress hormone levels, particularly cortisol. Anxiety activates the hypothalamic–pituitary–adrenal axis, resulting in immunoinflammatory dysregulation and elevated cortisol levels. While this response evolved to manage acute stress, chronic anxiety can cause metabolic changes that increase appetite and promote weight gain [43, 44]. Elevated cortisol levels enhance gluconeogenesis and promote fat storage, especially in the abdominal region, thereby contributing to weight gain. However, frequent stress responses, especially due to prolonged anxiety, can prevent complete recovery, keeping the body in a state of “stress-response hyperstimulation” or “hyperarousal” [45, 46]. This increased stress readiness often causes digestive issues, including reduced food intake due to discomfort, which leads to weight loss, as the body relies on fat stores with fewer calories. Consequently, there could be a two-way relationship between BMI and anxiety symptoms.

Subgroup analysis revealed that the impact of BMI on anxiety symptoms was more pronounced in females and individuals with lower education levels, indicating that gender differences and education levels may influence the BMI-anxiety relationship. Clinical and epidemiological studies have demonstrated gender differences in MDD, with women exhibiting a higher prevalence than men [47]. Women with MDD are also more likely to experience elevated anxiety levels and have a higher incidence of comorbid anxiety disorders [48]. In a cross-sectional study, approximately 70% of patients with depressive and anxiety disorders were females who exhibited increased psychological stress associated with obesity and heightened body dissatisfaction [49]. Moreover, estrogen modulates mood by influencing multiple neurotransmitter systems, including the noradrenergic, serotonergic, GABAergic, and dopaminergic pathways [50]. The regulatory effects of this hormone on neurotransmitters suggest a complex role in mood regulation. Conversely, vulnerability to “fat stigma” in everyday relationships among females exacerbates anxiety levels [51]. Several studies have demonstrated a significant inverse relationship between educational level and depression prevalence. Individuals with lower educational attainment are more likely to experience depressive symptoms [52, 53]. Individuals with higher educational levels typically have better job prospects, which can provide a sense of purpose and reduce stress, thereby lowering the risk of depression and anxiety. Education often correlates with socioeconomic status, and lower educational levels can lead to reduced income and limited access to resources, contributing to mental health challenges. Moreover, higher levels of education are linked to improved health literacy, empowering individuals to make informed health decisions, and access appropriate mental health services when needed.

This study has several limitations. First, the participant pool was restricted to Han Chinese individuals recruited from the psychiatric outpatient department of a single hospital in China, which may limit the applicability of the findings to other ethnic groups or healthcare settings. Future studies should include multicenter cohorts with diverse ethnic and clinical backgrounds to validate and expand upon these results. Second, while this study focused on patients with FEDN MDD, which minimized confounding treatment effects, it does not fully exclude the possibility that some participants might later be diagnosed with bipolar disorder, as their initial depressive symptoms may resemble MDD presentation. Third, the cross-sectional design of this study precludes the establishment of a causal relationship between BMI and anxiety symptoms in patients with MDD. Longitudinal studies are required to investigate this association further. Fourth, due to the small number of underweight participants (*n* = 10), they were combined with the normal BMI group to maintain statistical reliability. However, this classification may introduce potential bias, and future studies with larger sample sizes are required to clarify the impact of underweight status on anxiety symptoms in MDD. Fifth, patients with MDD in this study exhibited severe depression and anxiety, with HAMD-17 scores of ≥24 and total HAMA scores of at least 25. This limits the generalizability of our findings to less severely affected populations. Sixth, only BMI was used as an indicator of body mass, which, although often reflective of general obesity, does not capture the fat distribution. Additional measures, including skin-fold thickness measurements, can provide insights into regional obesity; neck circumference may reflect fat deposition in the upper body, waist circumference, waist-to-hip ratio, and the conicity index (waist-to-height ratio) can serve as indicators of abdominal fat deposition and could improve future studies. Final, this study did not consider certain confounding variables, including nutritional intake, physical activity, personality traits, socioeconomic factors, biological markers, and comorbid chronic diseases (for instance, hypertension and diabetes), as well as the medications used to treat them. These factors could potentially influence the relationship between BMI and anxiety symptoms. Incorporating them in future studies could provide a more comprehensive understanding of the pathophysiological mechanisms linking BMI and anxiety symptoms in MDD.

## 5. Conclusions

In this study, a nonlinear relationship between anxiety symptoms and BMI was identified in Chinese patients with FEDN MDD with an inflection point at 26.9 kg/m^2^. BMI exhibited a positive correlation with anxiety symptoms on the left side of the inflection point, whereas the correlation was not statistically significant on the right side. Notably, these associations were exacerbated in females and individuals with senior high school degrees. Furthermore, due to the study's limitations, including its cross-sectional design and failure to collect relevant data, the results should be considered preliminary and require validation in future longitudinal research.

## Figures and Tables

**Table 1:** Baseline characteristics of participants.

Variables	Total	BMI category			*p*-Value
		BMI < 24 kg/m^2^	24 ≤ BMI < 28 kg/m^2^	≥28 kg/m^2^	
*N*	1718	692	962	64	—
Age, years	34 (23, 45)	31 (22, 44)	35 (25, 46)	32 (24, 40)	0.005
Illness duration, months	5 (3, 8)	4.5 (3, 8)	5.5 (3, 8)	5 (3, 8)	0.009
Age of onset, years	34 (23, 45)	31 (22, 44)	35 (24.75, 46)	32 (24, 40)	0.005
Gender	—	—	—	—	0.219
Male, *n* (%)	588 (34.2%)	240 (34.7%)	320 (33.3%)	28 (43.8%)	—
Female, *n* (%)	1130 (65.8%)	452 (65.3%)	642 (66.7%)	36 (56.2%)	—
Education	—	—	—	—	0.326
Junior high school, *n* (%)	413 (24.0%)	155 (22.4%)	244 (25.4%)	14 (21.9%)	—
Senior high school, *n* (%)	760 (44.2%)	308 (44.5%)	428 (44.5%)	24 (37.5%)	—
College, *n* (%)	449 (26.1%)	183 (26.4%)	245 (25.4%)	21 (32.8%)	—
Postgraduate, *n* (%)	96 (5.6%)	46 (6.7%)	45 (4.7%)	5 (7.8%)	—
Marital status	—	—	—	—	<0.05
Single, *n* (%)	502 (29.2%)	226 (32.7%)	258 (26.8%)	18 (28.1%)	—
Married, *n* (%)	1216 (70.8%)	466 (67.3%)	704 (73.2%)	46 (71.9%)	—
HAMD score	30 (28, 32)	30 (28, 32)	30 (28, 32)	31 (29, 33)	0.059
HAMA score	21 (18, 23)	21 (18, 23)	21 (18, 23)	21 (19, 23)	0.447
TC, mmol/L	5.22 (4.46, 6.00)	5.12 (4.38, 5.89)	5.25 (4.48, 6.08)	5.50 (5.01, 6.25)	0.002
TG, mmol/L	1.97 (1.40, 2.77)	1.84 (1.34, 2.74)	2.06 (1.44, 2.78)	2.14 (1.36, 2.89)	0.038
HDL-C, mmol/L	1.23 (1.01, 1.42)	1.35 (1.03, 1.44)	1.22 (1.00, 1.39)	1.25 (0.91, 1.46)	0.057
LDL-C, mmol/L	2.97 (2.38, 3.52)	2.90 (2.32, 3.50)	2.97 (2.39, 3.50)	3.29 (2.43, 3.95)	0.059
FBG, mmol/L	5.34 (4.94, 5.80)	5.25 (4.87, 5.75)	5.40 (5.00, 5.86)	5.45 (4.97, 5.90)	<0.001
TSH, μIU/mL	4.91 (3.11, 6.67)	4.18 (2.43, 6.41)	5.24 (3.75, 6.77)	5.91 (5.23, 7.23)	<0.001
FT3, pmol/L	4.92 (4.38, 5.41)	4.87 (4.32, 5.39)	4.94 (4.42, 5.41)	5.01 (4.34, 5.25)	0.344
FT4, pmol/L	16.54 (14.38, 18.73)	16.42 (14.29, 18.77)	16.54 (14.48, 18.67)	17.65 (14.42, 19.37)	0.084
TgAb, IU/L	21.46 (14.43, 43.75)	22.13 (14.68, 44.57)	21.24 (14.13, 42.38)	21.89 (14.71, 53.90)	0.285
TPOAb, IU/L	17.43 (12.32, 34.74)	19.01 (12.71, 34.31)	16.73 (12,00, 35.15)	17.49 (12.31, 36.59)	0.065
Anxiety symptoms (*n*, %)	—	—	—	—	<0.01
No	1500 (87.3%)	623 (90.0%)	825 (85.8%)	52 (81.2%)	—
Yes	218 (12.7%)	69 (10.0%)	137 (14.2%)	12 (18.8%)	—

Abbreviations: BMI, body mass index (kg/m^2^); FBG, fasting blood glucose; FT3, free triiodothyronine (mmol/L); FT4, free thyroxine (mmol/L); HAMA, 14-item hamilton anxiety rating scale; HAMD, 17-item hamilton rating scale for depression; HDL-C, high-density lipoprotein cholesterol (mmol/L); LDL-C, low-density lipoprotein cholesterol (mmol/L); TC, total cholesterol (mmol/L); TG, triglyceride (mmol/L); TgAb, antithyroglobulin; TPOAb, thyroid peroxidases antibody; TSH, thyroid-stimulating hormone (mmol/L).

**Table 2:** Relationship between body mass index and anxiety symptoms in different models.

Group	*n*	Model 1		Model 2		Model 3	
		OR (95% CI)	*p*-Value	OR (95% CI)	*p*-Value	OR (95% CI)	*p*-Value
BMI (kg/m^2^)	1718	1.119 (1.039–1.205)	0.003	1.139 (1.047–1.241)	0.003	1.130 (1.039–1.229)	0.004
BMI category							
BMI < 24 kg/m^2^	692	Ref	1	Ref	1	Ref	1
24 ≤ BMI < 28 kg/m^2^	962	1.499 (1.102–2.039)	0.01	1.689 (1.168–2.444)	0.005	1.665 (1.159–2.392)	0.006
≥28 kg/m^2^	64	2.084 (1.061–4.093)	0.033	2.310 (1.048–5.093)	0.038	2.096 (0.958–4.586)	0.064

*Note:* Model 1: univariate analysis; Model 2: adjusted for age at onset, gender, duration of illness, education, HAMD, TC, TG, HDL-C, LDL-C, FBG, FT3, TGAb and TPOAb. Model 3: adjusted for duration of illness, HDL-C, TPOAb, TC and HAMD.

Abbreviations: BMI, body mass index; CI, confidence interval.

**Table 3:** Threshold effect of body mass index and anxiety symptoms using the piecewise logistic regression model.

Inflection point of BMI (kg/m^2^)	OR	95% CI	*p*-Value
Inflection point			
<26.9 kg/m^2^ (*n* = 1589)	1.167	1.055–1.296	0.003
≥26.9 kg/m^2^ (*n* = 129)	1.006	0.685–1.341	0.972
Log likelihood ratio test	—	—	0.039

*Note:* Adjusted for age at onset, gender, duration of illness, education, HAMD, TC, TG, HDL-C, LDL-C, FBG, FT3, TGAb, and TPOAb.

## Data Availability

The data that support the findings of this study are available on request from the corresponding author. The data are not publicly available due to privacy or ethical restrictions.
